# *Staphylococcus aureus* gene expression in a rat model of infective endocarditis

**DOI:** 10.1186/s13073-014-0093-3

**Published:** 2014-11-03

**Authors:** Frank Hanses, Christelle Roux, Paul M Dunman, Bernd Salzberger, Jean C Lee

**Affiliations:** Channing Laboratory, Department of Medicine, Brigham and Women’s Hospital and Harvard Medical School, Boston, MA 02115 USA; Department of Internal Medicine I, University Hospital Regensburg, Franz-Josef-Strauss Allee 11, Regensburg, 93049 Germany; Department of Microbiology and Immunology, University of Rochester, Rochester, NY 14642 USA

## Abstract

**Background:**

Diabetes mellitus is a frequent underlying comorbidity in patients with *Staphylococcus aureus* endocarditis, and it represents a risk factor for complications and a negative outcome. The pathogenesis of staphylococcal endocardial infections in diabetic hosts has been poorly characterized, and little is known about *S. aureus* gene expression in endocardial vegetations.

**Methods:**

We utilized a rat model of experimental *S. aureus* endocarditis to compare the pathogenesis of staphylococcal infection in diabetic and nondiabetic hosts and to study the global *S. aureus* transcriptome in endocardial vegetations in vivo.

**Results:**

Diabetic rats had higher levels of bacteremia and larger endocardial vegetations than nondiabetic control animals. Microarray analyses revealed that 61 *S. aureus* genes were upregulated in diabetic rats, and the majority of these bacterial genes were involved in amino acid and carbohydrate metabolism. When bacterial gene expression *in vivo* (diabetic or nondiabetic endocardial vegetations) was compared to *in vitro* growth conditions, higher *in vivo* expression of genes encoding toxins and proteases was observed. Additionally, genes involved in the production of adhesins, capsular polysaccharide, and siderophores, as well as in amino acid and carbohydrate transport and metabolism, were upregulated in endocardial vegetations. To test the contribution of selected upregulated genes to the pathogenesis of staphylococcal endocarditis, isogenic deletion mutants were utilized. A mutant defective in production of the siderophore staphyloferrin B was attenuated in the endocarditis model, whereas the virulence of a surface adhesin (Δ*sdrCDE)* mutant was similar to that of the parental *S. aureus* strain.

**Conclusions:**

Our results emphasize the relevance of diabetes mellitus as a risk factor for infectious endocarditis and provide a basis for understanding gene expression during staphylococcal infections in vivo.

**Electronic supplementary material:**

The online version of this article (doi:10.1186/s13073-014-0093-3) contains supplementary material, which is available to authorized users.

## Background

Infective endocarditis is an invasive human disease with an estimated incidence of 5 to 16 cases per 100,000 person-years [1,2] and an in-hospital mortality rate of 18% to 20% [3]. *Staphylococcus aureus* is the most frequent etiologic agent of endocarditis in developed countries [1,3] and is associated with an aggressive disease course, a poor outcome, and complications such as local destruction of valve tissue, septic emboli, and persistent bacteremia [3]. Antibiotic resistance is widespread among clinical *S. aureus* isolates, and bacteremia due to methicillin-resistant *S. aureus* (MRSA) often requires the use of expensive or less effective antibiotics.

Risk factors for endocarditis include injection drug use, prosthetic heart valves, structural heart defects, and comorbidities, such as diabetes [2,4,5]. Among patients with endocarditis, *S. aureus* is more frequently isolated from individuals with diabetes than those with no diabetes [6]. Moreover, patients with diabetes have higher mortality due to *S. aureus* endocarditis than patients without diabetes [7]. Although defects in the immune response to bacterial infections in patients with diabetes have been postulated [8], the mechanism(s) behind the increased susceptibility to invasive *S. aureus* infections remain elusive.

Several *S. aureus* virulence factors have been implicated in the pathogenesis of endocarditis. Fibronectin-binding protein A (FnBPA) and clumping factor A (ClfA) mediate staphylococcal adherence to endothelial cells [9,10]. Damage to cardiac valves results in exposure of the subendothelial matrix and deposition of fibrin and platelets at the site of endovascular injury. ClfA plays a role in *S. aureus* binding to platelets - an interaction that is critical to the induction of staphylococcal endocarditis [11]. Staphylococci recovered from rabbit endocardial vegetations are highly encapsulated [12]. Other factors shown to play a role in the pathogenesis of endocarditis include gene regulators such as *sar* and *agr* [13], alpha toxin [14], the sortase enzyme SrtA [15], and the proline permease PutP [16].

Although numerous studies have described in vivo expression of selected staphylococcal genes [17,18]), few have investigated overall patterns of bacterial gene expression in an infected host. Analyses of global gene expression patterns have focused primarily on in vitro conditions, and few descriptions of the *S. aureus* transcriptome during the course of an infection have been reported [19,20]. The *S. aureus* transcriptional profile during growth in broth culture correlates poorly with gene expression in mammalian infections, and further work to characterize *S. aureus* gene regulation in vivo is necessary [21].

Our goal was to assess the impact of concurrent diabetes mellitus on the course of endocarditis in a well-defined experimental infection model, to compare the transcriptional profile of *S. aureus* in established endocardial vegetations of diabetic and nondiabetic hosts versus growth *in vitro*, and to study the role of genes highly expressed *in vivo* as virulence factors in *S. aureus* endocarditis.

## Methods

### Bacteria and culture conditions

To evaluate differences in endocarditis disease severity and for microarray analyses, diabetic or nondiabetic rats were challenged with the MRSA strain COL. To establish the role of selected genes in the pathogenesis of *S. aureus* endocarditis, we utilized *S. aureus* strain Newman and its isogenic mutants. Newman Δ*sdrCDE* [22] was kindly provided by Dr Timothy Foster, and Newman Δ*sbnE* [23] was generously provided by Dr David Heinrichs. Staphylococci were cultivated in tryptic soy broth (TSB) to the mid-logarithmic phase, washed in phosphate-buffered saline (PBS), and diluted to yield an inoculum of approximately 3 × 10^4^ CFU/rat.

### Rat endocarditis model

This study was carried out in strict accordance with the recommendations in the Guide for the Care and Use of Laboratory Animals of the National Institutes of Health. All animal experiments were approved by the Harvard Medical School Standing Committee on Animals or by the local authorities in Regensburg. Diabetes was induced one day prior to surgery by injecting male Sprague-Dawley rats (approximately 200 g; Charles River Laboratories, Wilmington, MA, USA, and Sulzfeld, Germany) in the tail vein with streptozotocin (60 mg/kg); control rats received citrate buffer only [24]. Animals were considered diabetic if blood glucose levels exceeded 250 mg/dl after 24 h.

The rat model of catheter-induced *S. aureus* endocarditis was described previously [25]. Catheterized diabetic or nondiabetic rats were challenged intravenously with approximately 3 × 10^4^ CFU *S. aureus* 48 h after surgery. Heparinized blood was collected daily from each animal by tail vein puncture and plated quantitatively. Surviving rats were euthanized on day 3 after challenge. Catheter tips (about 2 cm) were removed, sonicated in PBS, and plated quantitatively. The kidneys, spleens, and aortic valve vegetations were weighed, homogenized, and cultured quantitatively. Homogenized vegetations were pelleted, suspended in RNAprotect Bacteria Reagent (Qiagen, Hilden, Germany) overnight at 4°C, and stored at -80°C.

### RNA isolation

Total RNA was prepared from homogenized vegetation pellets from single animals after digestion with 4 mg/ml proteinase K (Qiagen) for 30 min. *S. aureus* COL cells were lysed with 0.5 ml zirconia silica beads (Fisher Scientific, Waltham, MA, USA) in a dental amalgamator, and RNA was purified with the RNeasy Mini kit (Qiagen), treated with DNase I (Invitrogen, Grand Island, NY), and stored at -80°C. RNA integrity and absence of eukaryotic RNA were confirmed by denaturing gel electrophoresis. RNA from planktonic cultures was isolated from bacteria harvested from mid-logarithmic (5 h) or stationary phase (18 h) TSB cultures incubated at 37°C.

### Microarray analysis

Amplification of 10 ng RNA samples using ExpressArt Nanokits (Amsbio, Cambridge, MA) was performed according to the manufacturer’s recommendations for bacterial mRNA amplification, except that the final transcription and labeling step was performed using Enzo BioArray HighYield RNA Transcript Labeling kits (Farmingdale, NY, USA) to incorporate biotinylated ribonucleotides into final RNA products. Each biotinylated RNA sample (1 μg) was hybridized to Affymetrix *S. aureus* GeneChips (Santa Clara, CA, USA) following the manufacturer’s recommendations for antisense prokaryotic arrays. GeneChips were washed, stained, and scanned, as previously described [26]. Each condition (diabetic or nondiabetic rat and logarithmic or stationary phase culture) was analyzed in duplicate. GeneChip signal intensity values for each qualifier were then normalized, averaged, and analyzed using GeneSpring 6.2 software (Silicon Genetics, Redwood City, CA, USA), as described [26]. Genes were considered to be induced in a vegetation if they were determined to be present by Affymetrix algorithms in the endocarditis condition, and if they exhibited a greater than two-fold increase in RNA titer (Student’s *t*-test; *P* ≤0.05) compared to the corresponding planktonic growth condition. Genes were considered downregulated in the vegetations if they were present in either planktonic condition and had an expression level less than 50% of that observed in the corresponding planktonic growth condition (Student’s *t*-test; *P* ≤0.05). All GeneChip data have been deposited in the National Center for Biotechnology Information (NCBI) Gene Expression Omnibus (GEO) microarray repository under accession number [GEO:GSE62390].

### Quantitative PCR

cDNA was synthesized using the AffinityScript Multiple Temperature cDNA Synthesis Kit (Agilent Technologies, Santa Clara, CA, USA), 5 μl of hot-denatured DNA-free RNA, and 100 pmol of random hexamer primers. Products were precipitated in ethanol, resuspended in diethylpyrocarbonate-water, and stored at -20°C. Quantification was performed using a LightCycler 2.0 apparatus (Roche Diagnostics, Mannheim, Germany) and QuantiTect SYBR Green PCR Kits (Qiagen), using 2 μl of cDNA and 20 pmol of each primer. Primers (Additional file 1: Table S1) were synthesized by Eurofins MWG (Ebersberg, Germany).

### Statistical analysis

Student’s *t*-tests were used to compare gene expression data. Mann-Whitney *U* tests were performed to compare quantitative culture data, and the log rank test was used to compare survival distributions. A *P*-value of ≤0.05 was considered significant.

## Results

### Infective endocarditis was more severe in diabetic than nondiabetic rats

**Figure 1:**
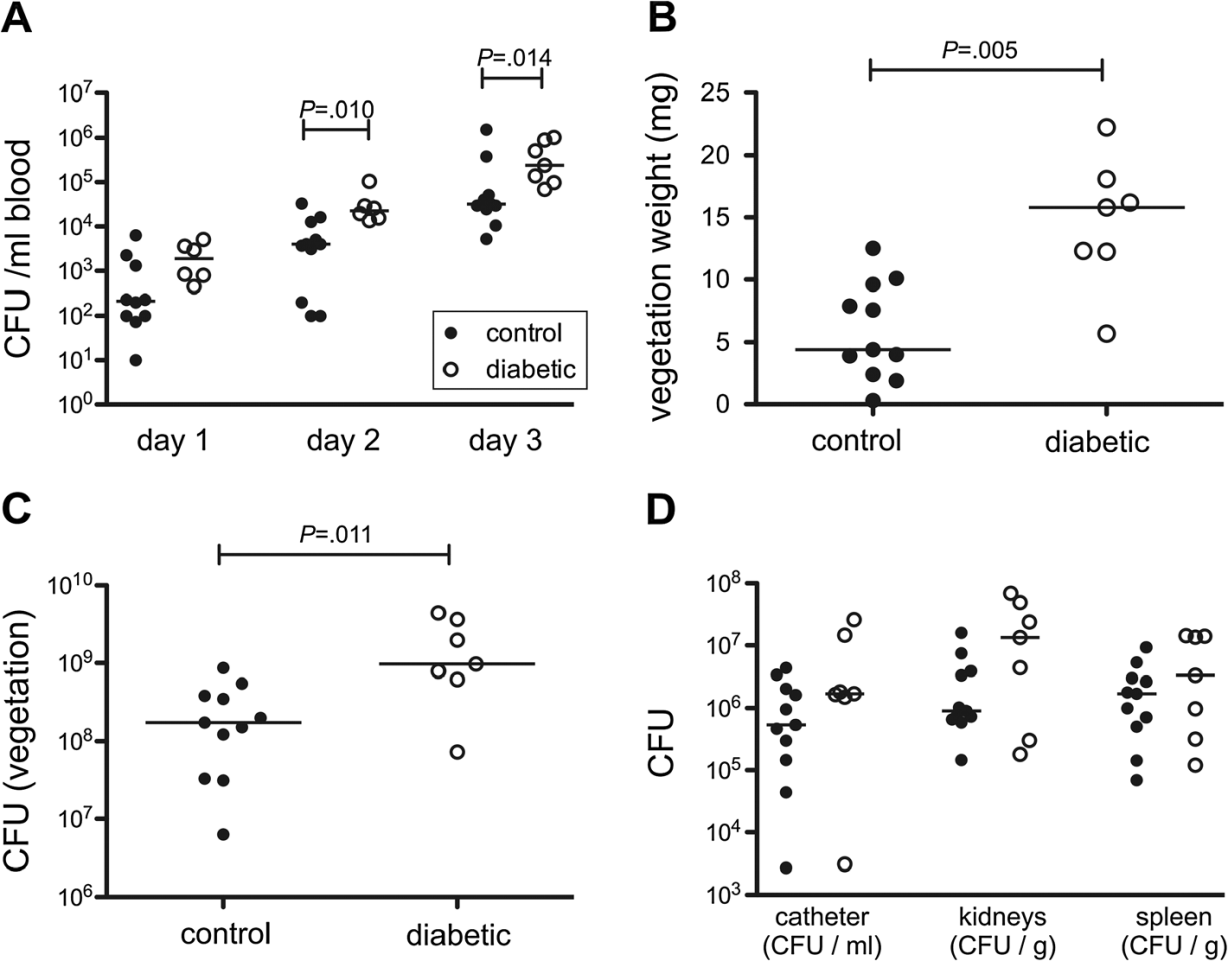
**Endocarditis was more severe in diabetic rats than in nondiabetic rats. (A)** Diabetic animals had significantly higher levels of bacteremia on day 2 and day 3 after bacterial challenge. **(B)** Diabetic animals had larger vegetations, and **(C)** the vegetations were associated with a significantly higher bacterial burden. **(D)** The bacterial burden in the kidneys, spleens, and catheters were not significantly different between diabetic and nondiabetic rats.

To assess the impact of diabetes on the pathogenesis of *S. aureus* endocarditis induced by strain COL, we compared the infection in rats with streptozotocin-induced diabetes to that of nondiabetic rats. All rats showed increasing bacteremia levels from days 1 to 3 (Figure 1A). Diabetic rats had significantly higher levels of bacteremia than nondiabetic rats on days 2 and 3 after inoculation (Figure 1A). Diabetic rats had larger endocardial vegetations (Figure 1B) with significantly more bacteria per vegetation (Figure 1C). We also observed trends toward elevated bacterial burdens in kidneys, spleens, and explanted catheters from diabetic rats compared with nondiabetic rats (Figure 1D), although these differences did not reach statistical significance.

### Differential *S. aureus* gene expression in diabetic and nondiabetic rats

We hypothesized that staphylococcal gene expression might be influenced by hyperglycemia in the diabetic host. Microarray analyses, however, indicated that overall gene expression was similar between the two groups of animals challenged with *S. aureus* COL. A total of 61 transcripts were more highly expressed under diabetic *in vivo* conditions (selected genes are presented in Table 1). Most of these genes were associated with metabolic processes such as amino acid and carbohydrate metabolism or cell homeostasis. LightCycler analyses confirmed higher expression of ATP synthase *atpA* (12.9 ±4.3 fold) and glyceraldehyde dehydrogenase *gapA* (10.6 ±2.1 fold) in vegetations from diabetic rats compared with nondiabetic rats. Only two genes (SACOL1895 and a 102 bp fragment, both with unknown functions) had significantly lower expression in the vegetations of diabetic compared with nondiabetic animals.

**Table 1:** **List of selected genes differentially expressed in diabetic and nondiabetic vegetations**

**Gene**	**Diabetic versus nondiabetic**	**Gene product**	**Functional group**
***acpS***	2.6	holo-(acyl-carrier-protein) synthase	Lipid transport and metabolism
***alr***	2.9	alanine racemase	Cell wall/membrane biogenesis
***argG***	10.0	argininosuccinate synthase	Amino acid transport and metabolism
***argH***	15.3	argininosuccinate lyase	Amino acid transport and metabolism
***atpA***	3.8	ATP synthase F1, alpha subunit	Energy production and conversion
***atpE***	2.4	ATP synthase F0, C subunit	Energy production and conversion
***atpH***	3.3	ATP synthase F1, delta subunit	Energy production and conversion
***cap5F***	2.5	capsular polysaccharide biosynthesis	Cell wall/membrane biogenesis
***cap5K***	3.5	capsular polysaccharide biosynthesis	Cell wall/membrane biogenesis
***ccrB***	2.0	cassette chromosome recombinase B	
***dnaE***	2.6	DNA polymerase III, alpha subunit	Nucleotide metabolism, replication, recombination
***fabD***	3.3	malonyl CoA-acyl carrier protein transacylase	Lipid transport and metabolism
***gapA***	4.4	glyceraldehyde 3-P dehydrogenase	Carbohydrate transport and metabolism
***geh***	2.3	glycerol ester hydrolase	Miscellaneous
***glpP***	2.2	glycerol uptake operon regulatory protein	Transcription, translation and post-translational modification
***glyS***	4.2	glycyl-tRNA synthetase	Transcription, translation and post-translational modification
***hemX***	2.2	hemX protein	Transcription, translation and post-translational mod.
***infB***	2.6	translation initiation factor IF-2	Transcription, translation and post-translational modification
***kdpE***	2.4	DNA-binding response regulator	Regulatory
***ligA***	2.3	DNA ligase, NAD-dependent	Nucleotide metabolism, replication, recombination
***menD***	2.1	carboxylic acid synthase	Coenzyme transport and metabolism
***moeA***	3.4	molybdopterin biosynthesis, putative	Coenzyme transport and metabolism
***nrdF***	2.7	ribonucleoside-diphosphate reductase 2	Nucleotide metabolism, replication, recombination
***pls***	3.7	plasmin-sensitive protein	Virulence
***recQ***	2.8	ATP-dependent DNA helicase	Nucleotide metabolism, replication, recombination
***rexA***	3.2	exonuclease	Nucleotide metabolism, replication, recombination
***ribD***	2.7	riboflavin biosynthesis protein	Coenzyme transport and metabolism
***ribE***	2.4	riboflavin synthase, alpha subunit	Coenzyme transport and metabolism
***ruvB***	2.2	Holliday junction DNA helicase	Nucleotide metabolism, replication, recombination
***sasA***	4.8	LPXTG cell wall surface protein	Virulence
***sirC***	3.1	iron ABC transporter, permease protein	Inorganic ion transport and metabolism
***ureC***	7.5	urease, alpha subunit	Amino acid transport and metabolism

### Differential *S. aureus* gene expression in endocardial vegetations

**Figure 2:**
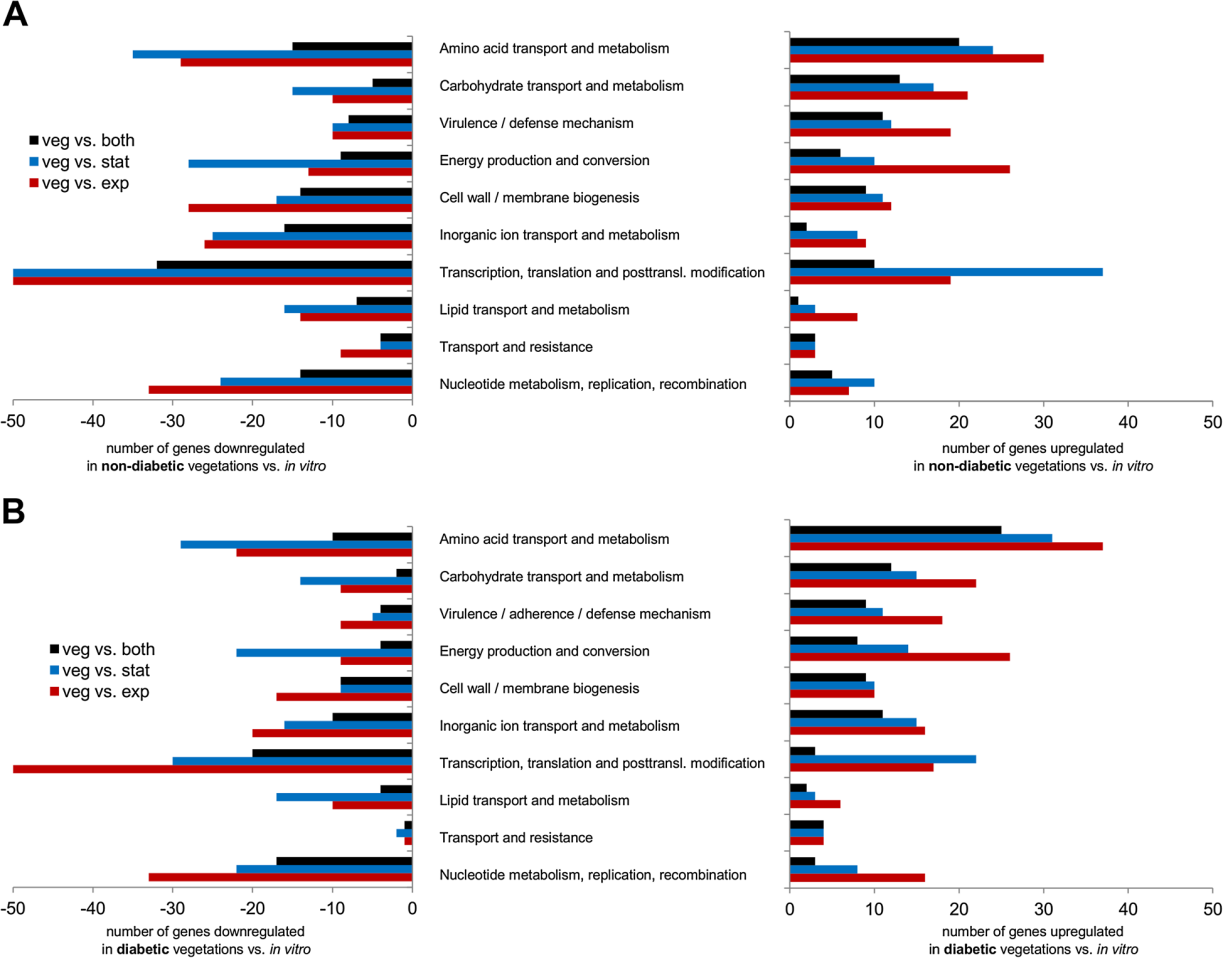
**Number of genes differentially expressed in endocardial vegetations from (A) nondiabetic and (B) diabetic rats.** Absolute number of genes down- (left) or upregulated (right) in endocardial vegetations compared with *in vitro* exponential (red bars), stationary (blue bars), or both growth phases (black bars). The four groups with the highest number of genes more highly expressed *in vivo* were amino acid transport and metabolism; carbohydrate transport and metabolism; virulence or immune evasion; and transcription, translation, and posttranslational modification.

To compare *S. aureus* gene expression *in vivo* versus *in vitro*, we isolated bacterial RNA from endocardial vegetations on day 3 post-challenge and from staphylococci in the logarithmic or stationary growth phase. Overall gene expression in diabetic and nondiabetic animals was similar, but differences between *in vivo* and *in vitro* growth conditions tended to be more pronounced in vegetations from diabetic hosts (Table 2). Microarray analyses revealed that 116 and 109 genes were more highly expressed in vegetations from nondiabetic and diabetic rats, respectively, compared with both planktonic conditions; 98 and 103 genes were more highly expressed *in vivo* compared with logarithmic phase cultures only; and 81 and 65 genes were more highly expressed *in vivo* compared with stationary phase cultures only. Additionally, we observed that 223 and 152 genes had reduced expression under *in vivo* conditions in nondiabetic and diabetic animals, respectively, compared with both planktonic conditions; 208 and 199 genes had lower expression *in vivo* compared to logarithmic phase cultures only; and 183 and 106 genes had lower expression *in vivo* compared to stationary phase cultures only. The largest group of genes differentially expressed in vegetations was genes of unknown function (hypothetical proteins). The next largest groups were genes involved in transcription, translation, and posttranslational modification; amino acid transport and metabolism; cell wall and membrane biogenesis; nucleotide metabolism and replication; carbohydrate transport and metabolism; and virulence or immune-evasion mechanisms (Figure 2). Numerous virulence-associated genes, such as toxins and proteases, were more highly expressed in endocardial vegetations than in planktonic cultures.

**Table 2:** **Selected genes that were differentially expressed in endocardial vegetations**

**Gene**		**Nondiabetic versus logarithmic phase**	**Vegetation versus stationary phase**	**Diabetic versus logarimthic phase**	**Vegetation versus stationary**	**Gene product**
**Virulence-associated genes**						
***hla***	SACOL1173	22.9	ns	17.5	ns	alpha-hemolysin precursor
***hld***	SACOL2022	38.5	ns	37.8	ns	delta-hemolysin
***hlgA***	SACOL2419	18.5	5.4	6.3	2.1	gamma-hemolysin, component A
***hlgB***	SACOL2421	11.8	2.7	ns	ns	gamma hemolysin, component B
***hlgC***	SACOL2422	16.5	2.4	ns	ns	gamma hemolysin, component C
***lukF***	SACOL2004	11.1	18.7	4.9	8.1	leukocidin F subunit
***lukS***	SACOL2006	11.9	14.7	6.4	8.0	leukocidin S subunit
***nuc***	SACOL0860	ns	ns	2.9	7.8	thermonuclease
***seb***	SACOL0907	8.0	ns	4.1	ns	staphylococcal enterotoxin B
***splB***	SACOL1868	10.7	5.7	ns.	ns	serine protease
***splC***	SACOL1867	ns	ns	3.4	2.5	serine protease
***splD***	SACOL1866	ns	ns	3.8	2.9	serine protease
***splE***	SACOL1865	28.4	11.6	16.9	7.5	serine protease
***aur***	SACOL2659	3.3	2.4	2.2	ns	zinc metalloproteinase aureolysin
***clpB***	SACOL0979	ns	ns	4.4	8.1	ATP-dependent protease
***ssaA***	SACOL2581	5.7	13.1	2.6	5.1	staphylococcal secretory antigen; staphyloxanthin biosynthesis protein
***sodA***	SACOL1610	-5.7	-4.5	-5.7	-4.5	superoxide dismutase
**Cell wall and capsule synthesis**						
***cap5A***	SACOL0136	8.5	2.8	8.8	3.0	capsular polysaccharide biosynthesis protein
***cap5B***	SACOL0137	7.3	2.6	7.4	2.6	capsular polysaccharide biosynthesis protein
***cap5D***	SACOL0139	6.3	2.2	7.4	3.8	capsular polysaccharide biosynthesis protein
***cap5E***	SACOL0140	13.4	10.2	9.1	6.8	capsular polysaccharide biosynthesis protein
***cap5F***	SACOL0141	5.3	3.6	ns	ns	capsular polysaccharide biosynthesis protein
***cap5G***	SACOL0142	4.3	2.5	6.6	4.0	capsular polysaccharide biosynthesis protein
***cap5I***	SACOL0144	3.3	ns	4.1	2.3	capsular polysaccharide biosynthesis protein
***cap5J***	SACOL0145	ns	ns	2.2	2.4	capsular polysaccharide biosynthesis protein
***dltA***	SACOL0935	-8.5	-2.9	-6.2	-2.1	D-alanine-ligase subunit 1
***dltB***	SACOL0936	-10.8	-3.4	-9.5	-2.8	D-alanyl-lipoteichoic acid biosynthesis protein
***dltC***	SACOL0937	-4.9	ns	-4.9	ns	D-alanine-ligase subunit 2
***dltD***	SACOL0938	-13.5	-3.5	-7.4	-2.0	D-alanine transfer protein
**Surface proteins**						
***clfA***	SACOL0856	5.8	ns	5.6	ns	clumping factor A
***clfB***	SACOL2652	ns	ns	ns	3.1	clumping factor B
***fnbA***	SACOL2511	3.4	2.7	ns	ns	fibronectin-binding protein A
***sasF***	SACOL2668	12.2	10.5	14.4	12.9	LPXTG cell wall surface anchor family protein
***sdrC***	SACOL0519	12.3	8.7	8.6	6.5	Ser-Asp repeat protein
***pls***	SACOL0050	-2.3	-3.3	ns	-4.3	surface protein
***sasA***	SACOL2676	ns	-9.8	2.2	-4.2	LPXTG cell wall surface anchor family protein
***spa***	SACOL0095	-6.3	-4.9	-5.7	-4.5	immunoglobulin G-binding protein A precursor
**Iron uptake and iron-regulated genes**						
***sbnA***	SACOL0112	ns	ns	48.9	22.2	siderophore biosynthesis protein
***sbnB***	SACOL0101	ns	ns	25.9	19.7	siderophore biosynthesis protein
***sbnD***	SACOL0103	ns	ns	18.4	17.1	siderophore biosynthesis protein
***sbnE***	SACOL0104	ns	ns	23.0	18.4	siderophore biosynthesis protein
***sbnG***	SACOL0106	ns	ns	21.6	25.2	siderophore biosynthesis protein
***sbnH***	SACOL0107	ns	ns	15.4	16.8	siderophore biosynthesis protein
***sbnI***	SACOL0108	ns	ns	23.5	30.0	siderophore biosynthesis protein
***sirR***	SACOL0691	2.3	ns	ns	ns	iron-dependent repressor
***sirB***	SACOL0098	2.9	ns	ns	ns	iron compound ABC transporter, permease
	SACOL2167	ns	ns	2.9	ns	iron complex transport substrate-binding protein
	SACOL2169	4.4	ns	4.2	ns	aerobactin biosynthesis protein
	SACOL2170	ns	ns	2.6	ns	major facilitator transporter
**Transport**						
***isdD***	SACOL1142	3.1	ns	2.3	ns	Heme ABC transporter
***dapF***	SACOL2479	18.2	18.9	15.2	15.6	diaminopimelate epimerase family protein
	SACOL2478	9.2	9.4	7.8	8.3	conserved hypothetical protein
	SACOL2477	17.6	12.8	20.9	15.1	conserved hypothetical protein
***opp1A***	SACOL2476	18.1	11.1	10.8	13.9	peptide ABC transporter
***opp1B***	SACOL2475	4.3	3.4	11.2	9.0	peptide ABC transporter, permease
***opp1C***	SACOL2474	15.9	12.9	30.4	24.0	peptide ABC transporter, permease
***opp1D***	SACOL2473	ns	ns	8.0	7.3	peptide ABC transporter ATP-binding protein
***opp1F***	SACOL2472	ns	ns	21.5	20.9	peptide ABC transporter ATP-binding protein
***norD***	SACOL2471	17.8	12.3	27.0	18.5	transporter, putative
***epiG***	SACOL1871	2.4	ns	2.6	ns	lantibiotic ABC transporter protein
***epiE***	SACOL1872	4.2	ns	4.4	ns	lantibiotic ABC transporter protein
***epiF***	SACOL1873	5.5	ns	5.7	ns	lantibiotic ABC transporter protein
***malE***	SACOL0193	ns	ns	3.1	2.5	maltose ABC transporter, maltose-binding
***malF***	SACOL0194	7.4	3.9	6.0	3.5	maltose ABC transporter, permease protein
***malK***	SACOL0192	14.7	14.7	6.2	6.8	maltose ABC transporter, ATP-binding protein
***opuD***	SACOL2176	2.0	2.4	ns	ns	osmoprotectant transporter, BCCT family
***tet38***	SACOL0122	21.2	12.2	13.1	8.9	tetracycline-resistance protein, putative
***ulaA***	SACOL0400	12.9	5.6	7.3	3.2	PTS system ascorbate-specific transporter
***uhpT***	SACOL0200	125	29	78.1	18.1	sugar phosphate antiporter
***kdpA***	SACOL2068	ns	ns	8.5	15.0	potassium-transporting ATPase, C subunit
***kdpB***	SACOL2067	ns	ns	9.0	13.3	potassium-transporting P-type ATPase, B unit
***norB***	SACOL1475	25.1	27.5	16.1	17.3	drug transporter, quinolone-resistance protein
	SACOL1476	24.8	22.4	18.7	18.6	transmembrane amino acid transporter
***ilvA***	SACOL1477	34.0	25.0	28.7	22.7	threonine dehydratase
***ald***	SACOL1478	42.3	24.0	31.2	17.6	alanine dehydrogenase
***mntH***	SACOL1114	-9.5	-6.3	-9.4	-6.3	Mn2+/Fe2+ transporter
	SACOL1115	-3.9	-4.0	-4.3	-4.4	hypothetical protein
***mntC***	SACOL0688	3.7	-5.8	3.5	-6.3	ABC transporter substrate-binding protein
***mntB***	SACOL0689	4.1	-5.7	4.5	-4.7	ABC transporter permease
***mntA***	SACOL0690	8.5	-2.7	5.6	-4.4	ABC transporter ATP-binding protein
	SACOL0157	-11.4	-12.0	-12.9	-12.8	conserved hypothetical protein
	SACOL0158	-6.8	-15.2	-6.1	-13.4	ABC transporter, ATP-binding protein
	SACOL0159	-6.9	-8.2	-5.0	-6.1	ABC transporter, permease protein
	SACOL0160	-4.9	-7.1	-5.5	-7.9	conserved hypothetical protein
**Metabolism**						
***adhE***	SACOL0135	22.9	22.3	30.9	31.1	alcohol dehydrogenase, iron-containing
***ald***	SACOL1478	42.3	24.0	31.2	17.6	alanine dehydrogenase
***arcB1***	SACOL1181	ns	ns	2.2	2.5	ornithine carbamoyltransferase
***arcC***	SACOL1182	21.8	15.3	12.9	9.9	carbamate kinase
***arcD***	SACOL1183	8.7	5.9	6.8	4.8	arginine/ornithine antiporter
***arcA***	SACOL2657	12.4	12.2	8.4	8.5	arginine deiminase
***arcB2***	SACOL2656	6.4	7.6	4.6	5.3	ornithine carbamoyltransferase
***arcD***	SACOL2655	30.1	43.5	27.6	43.1	arginine/ornithine antiporter
***argG***	SACOL0963	ns	ns	3.4	6.4	argininosuccinate synthase
***argH***	SACOL0964	ns	ns	5.4	13.3	argininosuccinate lyase
***deoB***	SACOL0124	4.0	2.2	4.4	2.3	phosphopentomutase
***deoD***	SACOL0121	7.1	7.8	8.9	10.0	purine nucleoside phosphorylase
***gltA***	SACOL1742	6.9	-6.7	4.8	-9.4	citrate synthase
***gntK***	SACOL2515	6.9	4.9	ns	ns	gluconokinase
***ilvA***	SACOL1477	34.0	25.0	28.7	22.7	threonine dehydratase
***nirB***	SACOL2398	21.8	19.6	12.8	11.9	nitrite reductase [NAD(P)H], large subunit
***nirD***	SACOL2397	8.1	6.1	4.7	3.9	nitrite reductase [NAD(P)H], small subunit
***pflA***	SACOL0205	94.7	125.7	116.5	157.6	pyruvate formate-lyase-activating enzyme
***pflB***	SACOL0204	44.7	46.9	59.9	65.6	formate acetyltransferase
***rbsK***	SACOL0253	6.9	2.5	4.9	ns.	ribokinase
	SACOL2396	19.0	14.3	7.4	6.1	uroporphyrin-III C-methyl transferase
	SACOL1476	24.8	22.4	18.7	18.6	amino acid permease
**Regulators**						
***agrA***	SACOL2026	ns	ns	4.8	ns	accessory gene regulator protein A
***agrC***	SACOL2025	-2.6	-3.3	4.5	-2.2	accessory gene regulator protein C
***agrD***	SACOL2024	2.3	-4.0	5.2	ns	accessory gene regulator protein D
***gntR***	SACOL2516	5.0	4.1	ns	ns	gluconate operon transcriptional repressor
***purR***	SACOL0539	ns	2.3	ns	ns	pur operon repressor
***rsbV***	SACOL2056	ns	3.6	ns	3.7	anti-anti-sigma factor RsbV
***rsbW***	SACOL2055	ns	2.7	ns	3.0	anti-sigma B factor
***sarA***	SACOL0672	ns	2.5	ns	2.1	transcriptional regulator
***sarS***	SACOL0096	-14.7	-7.2	-8.7	-4.2	transcriptional regulator
***sigB***	SACOL2054	ns	ns	ns	2.1	alternative sigma factor
***fur***	SACOL1541	ns	ns	-2.1	-3.1	transcriptional regulator, Fur family
***araC***	SACOL2378	-3.6	-2.7	ns	ns	transcriptional regulator, AraC family
***tetR***	SACOL2374	ns	ns	-2.0	-2.4	transcriptional regulator, TetR family
***vraR***	SACOL1942	-4.8	-3.2	-3.7	-2.5	DNA-binding response regulator
***vraS***	SACOL1943	-4.3	-2.8	-3.3	-2.2	sensor histidine kinase

ns, difference not significant.

A selection of *S. aureus* genes differentially expressed in endocardial vegetations compared to planktonic growth conditions *in vitro* is presented in Table 2. Among the virulence-associated upregulated genes were the alpha-, delta-, and gamma-hemolysins and the bi-component leukocidins LukS and LukF, which were more highly expressed under *in vivo* conditions than *in vitro* conditions. Likewise, genes encoding the metalloprotease aureolysin, the serine proteases SplBCDE, and the staphylococcal secretory antigen SsaA were all upregulated *in vivo*. Staphylococcal enterotoxin B (*seb*) showed higher expression in vegetations and in stationary growth phase compared with *S. aureus* in logarithmic growth phase. Neither *srtA* (encoding sortase A) nor *putP* (proline permease) showed differential expression *in vivo*. The superoxide dismutase *sodA* was significantly downregulated in endocardial vegetations compared with both *in vitro* conditions.

The capsule synthesis operon (*cap5A* to *cap5J*) was more highly expressed in vegetations than in either planktonic growth phases, whereas the *dltABCD* operon (encoding enzymes mediating D-alanylation of teichoic acids in the cell envelope) was downregulated *in vivo* compared with *in vitro*. Regarding the *S. aureus ica* locus that encodes poly-N-acetyl glucosamine, apparently COL does not synthesize this surface-associated polymer since it has a mutated *icaC* gene (SACOL2692). Most of the other *ica* genes were not differentially expressed *in vivo* and *in vitro*.

Among the staphylococcal surface proteins, clumping factor A (ClfA) and B (ClfB) genes were more highly expressed *in vivo* compared with logarithmic- or stationary growth-phase planktonic cultures, respectively. Fibronectin-binding protein A was significantly upregulated in the vegetations of nondiabetic rats. Genes encoding the LPXTG-anchored proteins SdrC (a beta-neurexin-binding protein) and SasF (associated with resistance to linoleic acid) were upregulated *in vivo* compared with both *in vitro* conditions. Of note, expression of both *spa* (encoding protein A) and *pls* (encoding a surface-associated plasmin-sensitive protein) were significantly lower *in vivo* than *in vitro*.

Regarding iron acquisition, we observed high *in vivo* expression of the *sbn* operon-encoding proteins required for the synthesis of the nonhydroxamate siderophore staphyloferrin B. *SbnABDEGHI* were significantly upregulated in vegetations from diabetic animals compared with logarithmic and stationary phase planktonic cultures. The same trend and magnitude of changes in expression were also observed in nondiabetic vegetations, but were not considered statistically significant. In addition, *sirB*, part of the *sirABC* operon encoding transport of staphyloferrin B into the bacterial cell [23,27], showed significant upregulation in nondiabetic rats with endocarditis. Some of the genes encoding the siderophore staphyloferrin A (SACOL2167, SACOL2169, and SACOL2170 [28]) were upregulated in endocardial vegetations (Table 2).

Several of the iron-regulated *S. aureus* genes were also involved in transport. Among the Isd genes that mediate heme uptake, only *isdD* (a heme ABC transporter) was consistently upregulated *in vivo* and only versus logarithmic phase *S. aureus.* Other iron-regulated genes with significantly higher expression *in vivo* included the oligopeptide permease and *norD* genes of the *opp1ABCDFnorD* operon [29]. Numerous genes encoding transporters were highly expressed by *S. aureus* in endocardial vegetations. The maltose transport (*malEFK*) genes were almost uniformly upregulated *in vivo* by staphylococci recovered from infected vegetations of nondiabetic and diabetic rats. Similarly, *kdpAB* genes encoding a potassium-transporting ATPase were upregulated in diabetic vegetations compared to both *in vitro* growth conditions. Other upregulated transporters include *epiGEF*, involved in lantibiotic transport, and *tet38* and *norB*, mediating drug resistance*.* Of note, *mntH* (a Mn^2+^/Fe^2+^ transporter) was downregulated *in vivo* compared with both planktonic conditions, whereas the *mntABC* operon (involved in Mn^2+^ uptake [30]) was upregulated compared with logarithmic phase *S. aureus* and downregulated compared with stationary phase cells. A large uncharacterized transporter operon (SACOL2471-2479) was upregulated *in vivo*, whereas the SACOL0157 to SACOL0160 operon had significantly lower expression levels in vegetations than in planktonic cultures.

Genes associated with metabolic pathways and more highly expressed in vegetations were numerous and included an alcohol dehydrogenase (*adhE*), nitrite reductase (*nirBD*), and genes implicated in arginine (*argGH* and two *arc* operons) and pyruvate (*pflAB*) metabolism.

Transcription of a few staphylococcal regulatory genes was enhanced *in vivo*. The transcriptional regulator GntR showed higher expression *in vivo* in nondiabetic rats. By contrast, genes encoding the staphylococcal accessory regulator SarS, the iron-regulated repressor Fur, the regulators VraR and VraS, and the transcriptional regulator AraC had lower expression *in vivo* than *in vitro* (Table 2). The *agr* locus did not show consistent changes *in vivo* and *in vitro* (Table 2), although in diabetic rats the locus was upregulated compared with *in vitro* logarithmic phase cultures.

### Confirmation of upregulated genes in endocardial vegetations by real-time PCR

We used quantitative PCR to confirm differential expression of selected genes *in vivo*. Genes were selected based on upregulation in microarray analysis, being a potential virulence factor, and not having been implicated in the pathogenesis of endocarditis previously. *In vitro* or *in vivo* expression of *sbnC*, *sdrC*, and *splB* was thus quantified using the LightCycler real-time PCR system. Although the magnitude of the differences in gene expression varied, we observed an overall good correlation between gene expression measured by microarray and LightCycler. All three genes tested showed a trend towards higher gene expression levels *in vivo*, but only *splB* showed significantly higher gene expression in endocardial vegetations compared with both planktonic growth phases (60.4-fold upregulation versus logarithmic growth phase, *P* <0.01; and 8.6-fold versus stationary growth phase, *P* = 0.03). Expression levels of *sbnC* and *sdrC* in endocardial vegetations were significantly higher compared with logarithmic growth phase *S. aureus* (502.3-fold for *sbnC*, *P* <0.01; and 25.1-fold for *sdrC*, *P* <0.01).

### Role of selected genes in the pathogenesis of *S. aureus* endocarditis

**Figure 3:**
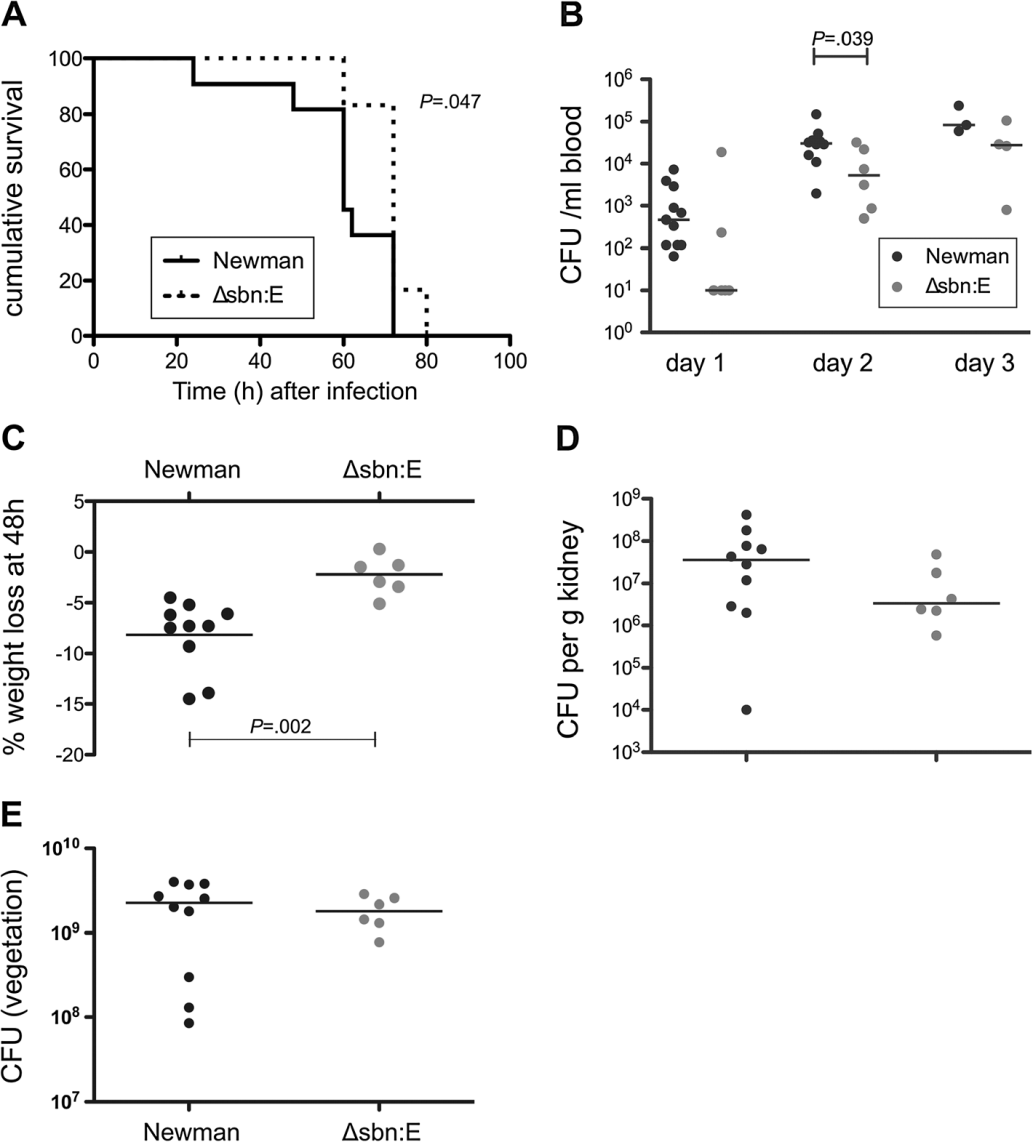
**Comparative virulence of strain Newman and its isogenic** ***sbnE*** **deletion mutant. (A)** Compared to rats infected with strain Newman, rats infected with the *sbnE* mutant (defective in the synthesis of the siderophore staphyloferrin **B** lived significantly longer than rats infected with the parental strain, **(B)** had significantly lower levels of bacteremia on day 2, and **(C)** lost significantly less weight 48 h after bacterial inoculation. **(D)** Bacterial burdens in the kidneys and **(E)** vegetations were not significantly different between wild-type and mutant strain.

To assess the contribution of selected genes preferentially expressed *in vivo* to the pathogenesis of *S. aureus* endocarditis, we performed virulence studies in the nondiabetic rat endocarditis model. We chose genes that had not previously been implicated as important in endocarditis - the *sbn* locus (encoding staphyloferrin B) and the *sdrCDE* locus (involved in fibrinogen-mediated *S. aureus* adherence to platelets under *in vitro* shear flow conditions [31]). Rats infected with Newman Δ*sbnE* survived longer than rats infected with the parental isolate (Figure 3A; *P* = 0.047, log-rank analysis), and they had lower bacteremia levels at all time points, although the differences reached significance only on day 2 (*P =* 0.039; Figure 3B). Likewise, rats infected with strain Newman experienced greater weight loss (*P =* 0.002) than rats infected with the *sbnE* mutant (Figure 3C). No significant differences were observed in the bacterial burden in kidneys (Figure 3D) or vegetations (Figure 3E) from rats infected with Newman or the Δ*sbnE* mutant. By contrast, rats infected with strain Newman or the Δ*sdrCDE* mutant had similar survival times (not shown), bacteremia levels (Additional file 1: Figure S1A), and weight loss (Additional file 1: Figure S1B). Likewise, the bacterial burdens in the kidneys (Additional file 1: Figure S1C) and endocardial vegetations (Additional file 1: Figure S1D) were comparable for wild-type and Δ*sdrCDE* strains.

## Discussion

Patients with diabetes have a higher risk of staphylococcal infections than those without diabetes, and patients with diabetes who develop *S. aureus* infective endocarditis are likely to experience a poor outcome [2,5]. We have demonstrated in an animal model of *S. aureus* endocarditis that diabetes is associated with a more severe disease course, as reflected by higher levels of bacteremia and larger endocardial vegetations. We used the vegetations recovered from infected rats on infection day 3 to investigate the *in vivo* transcriptome of *S. aureus* in the setting of an acute infection*.* This time point allowed maximal recovery of organisms multiplying within the host and allowed us to recover sufficient bacterial RNA for analysis.

Characterization of gene regulation and gene expression under *in vivo* conditions is a prerequisite for alternative treatment approaches, including the development of new drug targets and vaccination strategies. The *S. aureus* transcriptome under *in vivo* conditions has not been well characterized. Global *S. aureus* gene expression *in vitro* has been investigated after *in vitro* exposure to microbicides [32], in response to mild acid [33], during growth in biofilm [34], in blood [35], and after phagocytosis [36]. Changes in *S. aureus* gene expression during early adaptation to the mouse lung have been described [19], and Date *et al*. recently reported global *S. aureus* gene expression in human abscesses and infected murine kidneys [20]. Our study is the first description of the *S. aureus* transcriptome during an established endocardial infection. We used rich medium (TSB) as an *in vitro* comparator condition, although minimal medium such as Roswell Park Memorial Institute medium may better reflect iron-limited conditions *in vivo*. The choice of TSB as the *in vitro* comparator medium may explain some of the differences in gene expression reported (for example, iron-rich medium versus iron-depleted *in vivo* conditions). However, one of the aims of our study was to compare the gene expression pattern observed in endocarditis vegetations to gene expression found during growth under standard laboratory conditions. Our approach thus ensures comparability to previously published *in vitro* data (including previous microarray data, for example [26,36-38]). It also highlights the important contribution of *in vivo* conditions (for example, iron starvation) to *S. aureus* gene expression during an infection. An additional limitation of our study is the lack of a glucose-supplemented *in vitro* control arm. However, the fact that we did not observe significant differences in *S. aureus* virulence gene expression between nondiabetic and diabetic rats makes this additional control less important.

*S. aureus* COL is a well-defined and sequenced MRSA strain, which we used for the experiments with diabetic versus nondiabetic rats and the microarray analyses. Moreover, all gene expression studies and subsequent analyses were performed with strain COL. A related methicillin-sensitive *S. aureus* strain (Newman [39]) was used for the single gene knockout studies because of the availability of relevant mutant strains. Mutations in strains Newman and COL have been described [40-42], and both strains are members of clonal complex 8 and readily produce endocarditis in rats.

We observed that more than 100 genes were upregulated in endocardial vegetations compared with *in vitro* cultures. However, the number of genes significantly downregulated *in vivo* was approximately two-fold higher than the number of genes upregulated *in vivo*. This may be because *in vivo S. aureus* gene expression was analyzed from vegetations three days after bacterial challenge. Date *et al*. [20] reported similar gene expression patterns in human abscesses and infected murine kidneys for some *S. aureus* genes, such as the serine proteases *spl*, gamma-hemolysin, and the *opp1* operon. Other genes with higher expression levels in abscesses, such as the *isd* genes, however, were not differentially expressed in endocardial vegetations in our study.

A majority of the genes that were differentially expressed in endocardial vegetations have not yet been assigned a function or are classified as hypothetical proteins. ClfA is a critical virulence factor in *S. aureus* endocarditis [10] and *clfA* transcript levels have been reported to increase after the late logarithmic growth phase *in vitro* [43]. In our study, *clfA* was upregulated *in vivo* versus logarithmic growth phase cells, whereas fibronectin-binding protein A was found to be significantly upregulated in nondiabetic vegetations only. Both are likely to be critical for initiation of infection, as has been described in previous studies [44,45]. Although acapsular strains provoke endocarditis at lower inocula than encapsulated isolates [46], *S. aureus* recovered from vegetations produce large quantities of capsular polysaccharides [12], consistent with our observation that the *cap5* genes were upregulated in established vegetations. Capsule production enhances bacteremia *in vivo* and renders the bacterium resistant to uptake and killing by neutrophils [47]. Moreover, active and/or passive immunization strategies targeting capsular polysaccharides were able to protect rodents against *S. aureus* bacteremia [48] and endocarditis [25]. In contrast to the *cap5* genes, the *dlt* operon was downregulated in endocardial vegetations. The *dlt* genes encode proteins mediating D-alanylation of lipoteichoic and wall teichoic acids, and transcription of the *dlt* genes is repressed in the presence of cations such as magnesium salt [49]. Higher *dlt* expression has been associated with daptomycin resistance [50], and the observation that *dlt* is downregulated in endocardial vegetations could thus support clinical and preclinical data on the use of daptomycin in staphylococcal endocarditis [51,52].

Other virulence factors upregulated *in vivo* include exotoxins (hemolysins and leukotoxins) and proteases. The serine protease genes *splA* to *splE* are located on a pathogenicity island [39], and these proteases play a role in the degradation and inactivation of antibacterial peptides [53]. Both Spl proteases and aureolysin (*aur*) are associated with detachment of *S. aureus* from established biofilms [54] and may promote bacteremia by dispersing staphylococci from endocardial vegetations. Exotoxins upregulated in endocardial vegetations include two-component pore-forming cytolysins such as gamma-hemolysin and LukFS that are able to lyse erythrocytes and leukocytes [55]. Other factors previously implicated in the pathogenesis of endocarditis, such as SrtA [15] and PutP [16], were not differentially expressed in endocardial vegetations compared with planktonic conditions.

To acquire iron from the host organism, *S. aureus* synthesizes two major nonhydroxamate siderophores (staphyloferrin A and staphyloferrin B [28,56]), as well as proteins that mediate the import and utilization of iron bound to heme or transferrin [57]. Although heme was identified as a preferred iron source for *S. aureus* [57], we found that genes for neither the iron surface determinant *(isd*) nor the heme transport system (*hts*) were significantly upregulated in endocardial vegetations (with the exception of *isdD* compared with exponential phase cultures). By contrast, the staphyloferrin B synthesis operon (*sbnA* to *sbnI*) was preferentially upregulated *in vivo*. There was also a trend towards higher expression *in vivo* of the *fhuCBG* operon encoding an ABC transporter for iron(III)-hydroxamates [58]. However, *fhuD1* and *fhuD2*, encoding iron(III)-hydroxamate-binding lipoproteins, were not upregulated in endocarditis (not shown). The observed differences in iron uptake systems may in part be attributable to different experimental conditions and the animal models employed (for example [59]).

Dale *et al*. [23] constructed a Newman mutant (*ΔsbnE*) deficient in staphyloferrin B synthesis, and introduction of *sbnE* on a plasmid complemented the inability of the mutant to produce the siderophore under iron-limiting conditions. The Newman *ΔsbnE* mutant was compromised in the *S. aureus* renal abscess model [23]. This mutant was attenuated in the endocarditis model because rats inoculated with the mutant showed lower bacteremia levels, reduced weight loss, and increased survival compared with animals challenged with the parental strain. Although the reduction in virulence was modest, this can likely be attributed to the multitude of virulence factors involved in the pathogenesis of staphylococcal endocarditis. Thus, staphyloferrin B appears to promote staphylococcal virulence in abscesses and endocarditis.

Genes encoding multiple transporters were highly expressed in endocardial vegetations, including amino acid permeases, the lantibiotic transporter proteins EpiEFG, a maltose transporter, a potassium ATPase, the *norD-opp1* operon, and genes associated with drug resistance, such as *tet38* and *norB*. NorB promoted bacterial survival in a subcutaneous abscess model [17], and has broad substrate specificity. Upregulation of *norB* under conditions of low pH or reduced aeration has been reported [60]. Similarly, the efflux pump transporter NorD is iron-regulated and highly expressed in staphylococcal abscesses [29]. *NorD* is part of an operon with five upstream oligopeptide permease genes (*opp1ABCDF*), and all were significantly upregulated in endocardial vegetations. NorB and NorD efflux pumps may eliminate toxic metabolites or antibacterial factors produced by the host *in vivo*, although their substrates remain to be defined.

Among the surface molecules with higher expression in endocardial vegetations were the LPXTG-containing protein SasF, and the β-neurexin-binding protein SdrC. Genes within the *sdr* region (*sdrCDE)* have been reported to be differentially transcribed [61], and *sdr* genes other than *sdrC* were not upregulated in endocardial vegetations. SdrC could mediate initial contact with host cells or cell adhesion molecules, such as β-neurexin [62], although our experimental endocarditis data indicate that it was not critical for the establishment of endocarditis. SdrC may play a role in complications of infectious endocarditis, such as metastatic seeding to neuronal tissues.

The largest group of genes differentially expressed *in vivo* were involved in amino acid transport and metabolism. The chromosomal arginine deiminase pathway (*arcABCD*) was strongly upregulated in endocardial vegetations. Arginine metabolism may serve to counter acidification or as an energy source under anaerobic conditions [63]. The latter is supported by the fact that nitrate reductase (NirBD) and pyruvate formate lyase (PflAB [64]) were also upregulated in endocardial vegetations. Expression of the superoxide dismutase gene s*odA,* which is induced under microaerophilic conditions [65], was reduced in endocardial vegetations. Although staphylococcal vegetations grow in the oxygen-rich bloodstream at the aortic valve, the microenvironment within the vegetation may be oxygen-starved. Of note, we also observed differential gene expression of several gene clusters with yet unknown function (for example, upregulation of SACOL2477 to SACOL2479 and downregulation of SACOL0157 to SACOL0160 in endocardial vegetations). Their role in staphylococcal infections and contribution to the pathogenesis of endocarditis in particular remains to be determined.

*S. aureus* gene expression is controlled by a complex network of regulatory proteins. In a previous study, expression of *S. aureus agr* in rabbit endocardial vegetations was shown to increase over time and to correlate with bacterial densities in vegetations [66]. We observed that the *agr* operon was only consistently upregulated in endocardial vegetations, compared to cultures harvested from the logarithmic growth phase, in diabetic rats. The *S. aureus fur* regulator, which represses genes involved in iron acquisition, cytolysins, and immunomodulatory proteins [67], also had lower expression in endocardial vegetations than *in vitro*, as did the transcriptional regulator *sarS*. Differential expression of other regulatory genes included downregulation of *vraSR* (involved in resistance to cell wall active antibiotics and antimicrobial peptides [68]) and the *araC* family regulator (linking environmental chemical signals and virulence factors [69]), and upregulation of the gluconate repressor operon *gntR*. The *saeS* gene was significantly downregulated *in vivo* in the vegetations of diabetic rats.

With regard to genes differentially expressed in endocardial vegetations from diabetic and nondiabetic rats, we found 61 genes with higher expression levels in vegetations from diabetic animals. Although bacterial densities in the vegetations from both groups were similar (not shown), vegetations from diabetic animals were significantly larger and contained more bacteria than their nondiabetic counterparts. The composition or size of these vegetations may have an influence on staphylococcal gene expression in endocarditis. Of note, several of the differentially expressed genes with high upregulation (*argG, argH, glyS, ureC*) were previously reported to be upregulated in biofilms versus planktonic cultures [26]. Other genes found to be upregulated in diabetic vegetations (*gapA, hemX, nrdF*) were demonstrated to be under the control of glucose and CcpA [70]. In summary, we cannot exclude that differences in gene expression observed between diabetic and nondiabetic rats with endocarditis reflect differences in the size of the vegetations involved (possibly attributable to an altered innate immune response in diabetic hosts). However, there is also evidence that *S. aureus* may sense (and possibly benefit from) increased glucose levels in hosts with diabetes mellitus.

## Conclusions

We have demonstrated that diabetes mellitus leads to higher bacterial burdens in an experimental model of *S. aureus* endocarditis. Gene expression patterns in endocardial vegetations differed from those observed during *in vitro* growth, with overall downregulation of transcription. Endocarditis promoted higher expression of genes encoding toxins, surface proteins, and enzymes involved in capsule biosynthesis, iron homeostasis, and glucose and amino acid metabolism. Our findings indicate that gene expression of the staphyloferrin B operon is upregulated in endocarditis, and that production of this siderophore promoted staphylococcal virulence in this infection model. These results may form a basis for future analyses of specific genes, regulators, and pathways that are critical in endocarditis and other infections. Our findings may thus contribute to a better understanding of *S. aureus* pathogenesis *in vivo* and lead to alternative approaches in prevention or treatment of staphylococcal endocarditis.

## Additional file

Additional file 1: Figure S1. Comparative virulence of strain Newman and its isogenic *sdrCDE* deletion mutant. **(A)** Infection with the mutant strain led to comparable levels of bacteremia, **(B)** weight loss at 48 h, **(C)** bacterial burden in the kidneys and **(D)** endocardial vegetations compared to infection with the parental strain. **Table S1.** Primers used for real-time PCR.
