# Supplementary material for: Staphylococcus aureus gene expression in a rat model of infective endocarditis
Source: Genome Med. 2014 Nov 3;6(10):93. doi: 10.1186/s13073-014-0093-3 (PMC4228149; doi:10.1186/s13073-014-0093-3)
Supplement: Additional file 1: Figure S1. — Comparative virulence of strain Newman and its isogenic sdrCDE deletion mutant. (A) Infection with the mutant strain led to comparable levels of bacteremia, (B) weight loss at 48 h, (C) bacterial burden in the kidneys and (D) endocardial vegetations compared to infection with the parental strain. Table S1. Primers used for real-time PCR. [file 13073_2014_93_MOESM1_ESM.docx]

# Supplementary data


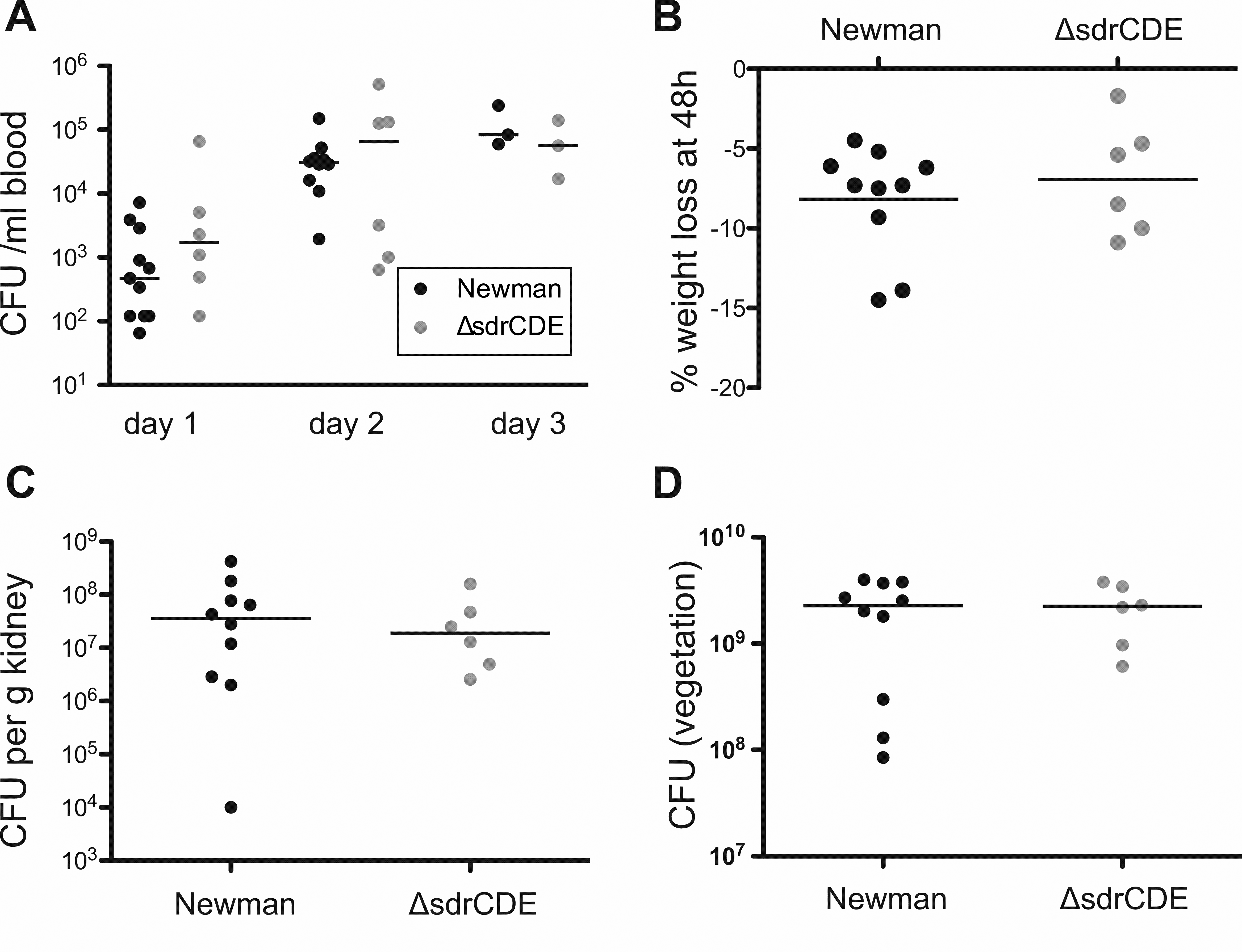


FIG S1 Comparative virulence of strain Newman and its isogenic *sdrCDE* deletion mutant. (A) Infection with the mutant strain led to comparable levels of bacteremia, (B) weight loss at 48 h, (C) bacterial burden in the kidneys and (D) endocardial vegetations compared to infection with the parental strain.

**TABLE S1.** Primers used for real-time PCR

| **Gene** |  | **Sequence** |
| --- | --- | --- |
| *atpA* | forward | CCAGGTCGTGAAGCATACCCAGG |
| *atpA* | reverse | CAGATTGTCCGGCATTAATCGCTGG |
| *gapA* | forward | GGTAGTTGATGGTGGTTTCCGCG |
| *gapA* | reverse | CGCCTGCTTCAATATGAGCTTGTGC |
| *gyrB* | forward | CGACTTCAGAGAGAGGTTTGCACCAT |
| *gyrB* | reverse | TCCGCCACCGCCAAATTTACCA |
| *rrsA* | forward | ATGTTAGCGGCGGACGGGTG |
| *rrsA* | reverse | CGATCACCCTCTCAGGTCGGCT |
| *sbnC* | forward | AGTCGTCCCGCATCAATCACAAAATT |
| *sbnC* | reverse | TGATGGTCATTCAGTTCGTATACGGCA |
| *sdrC* | forward | GGGTTAAGTGGTCATGAAGCTAAAGCG |
| *sdrC* | reverse | GGCTGATCTGCAGTTGCAGTTTGC |
| *splB* | forward | TGGTTGAGGAAGTACAACAAACTGCCA |
| *splB* | reverse | TGTGCAGTAATACGATCGCCCACT |
